# Histone deacetylase Hos2 regulates protein expression noise by potentially modulating the protein translation machinery

**DOI:** 10.1093/nar/gkae432

**Published:** 2024-05-23

**Authors:** Wei-Han Lin, Florica J G Opoc, Chia-Wei Liao, Kevin R Roy, Lars M Steinmetz, Jun-Yi Leu

**Affiliations:** Doctoral Program in Microbial Genomics, National Chung Hsing University and Academia Sinica, Taiwan; Institute of Molecular Biology, Academia Sinica, Taipei 115, Taiwan; Institute of Molecular Biology, Academia Sinica, Taipei 115, Taiwan; Institute of Molecular Biology, Academia Sinica, Taipei 115, Taiwan; Stanford Genome Technology Center, Stanford University, Palo Alto, CA 94304, USA; Department of Genetics, Stanford University School of Medicine, Stanford, CA 94305, USA; Stanford Genome Technology Center, Stanford University, Palo Alto, CA 94304, USA; Department of Genetics, Stanford University School of Medicine, Stanford, CA 94305, USA; European Molecular Biology Laboratory (EMBL), Genome Biology Unit, Heidelberg 69117, Germany; Doctoral Program in Microbial Genomics, National Chung Hsing University and Academia Sinica, Taiwan; Institute of Molecular Biology, Academia Sinica, Taipei 115, Taiwan

## Abstract

Non-genetic variations derived from expression noise at transcript or protein levels can result in cell-to-cell heterogeneity within an isogenic population. Although cells have developed strategies to reduce noise in some cellular functions, this heterogeneity can also facilitate varying levels of regulation and provide evolutionary benefits in specific environments. Despite several general characteristics of cellular noise having been revealed, the detailed molecular pathways underlying noise regulation remain elusive. Here, we established a dual-fluorescent reporter system in *Saccharomyces cerevisiae* and performed experimental evolution to search for mutations that increase expression noise. By analyzing evolved cells using bulk segregant analysis coupled with whole-genome sequencing, we identified the histone deacetylase Hos2 as a negative noise regulator. A *hos2* mutant down-regulated multiple ribosomal protein genes and exhibited partially compromised protein translation, indicating that Hos2 may regulate protein expression noise by modulating the translation machinery. Treating cells with translation inhibitors or introducing mutations into several Hos2-regulated ribosomal protein genes—*RPS9A*, *RPS28B* and *RPL42A*—enhanced protein expression noise. Our study provides an effective strategy for identifying noise regulators and also sheds light on how cells regulate non-genetic variation through protein translation.

## Graphical Abstract

**Graphical Abstract:**
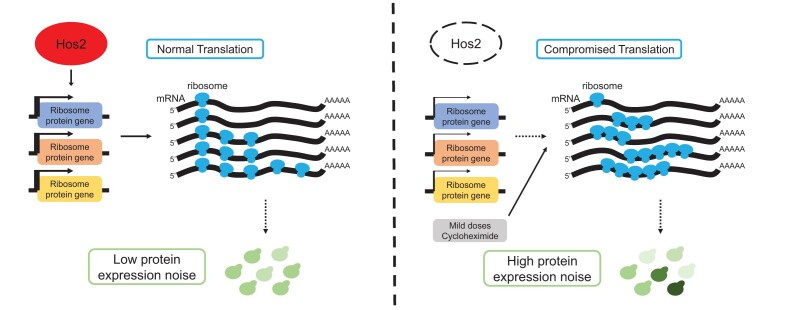

## Introduction

Phenotypic differences among individuals in a population can be attributed to both genetic and non-genetic effects. Even in a genetically identical population in a homogeneous environment, cell-to-cell phenotypic diversity can still arise by non-genetic variation, such as expression noise, which is derived from heterogeneity in transcript or protein levels (1). Random fluctuations in the expression levels of individual mRNAs or proteins are inevitable (2), which are attributable to the intrinsically stochastic nature of molecular processes in gene expression, such as transcriptional dynamics at promoter sites, chromatin modifications, mRNA degradation, translation rates, and post-translational regulation (3–5). Excessive expression noise can limit the accuracy of cellular processes and be potentially deleterious (6). To prevent the harmful effects caused by stochastic variation, processes have evolved to minimize and compensate for noise, especially for genes critical to cell viability (6–9). Essential genes in yeast tend to be chromosomally clustered and enriched in noiseless genomic regions, indicating that the levels and expression timing of these gene products need to be precisely controlled (10,11).

Nevertheless, expression noise in a clonal population can also endow the advantage of rapid responsiveness to fluctuating environmental stimuli (3). In microbial cells, levels of preexisting cell-to-cell heterogeneity can affect the fitness of a population upon encountering sudden environmental stress (12). For instance, in a clonal population subjected to H_2_O_2_ treatments, yeast cells with high levels of Tdh2, a protein involved in oxidative stress resistance during the stationary growth phase (13), exhibited higher survival rates than those with low Tdh2 levels (14). This observation indicates that expression noise can provide benefits in a subpopulation of cells during a stress response, thereby ensuring population survival (14). Interestingly, stress-responsive genes were found to be more noisy than essential genes in yeast (9,11), indicating a bet-hedging (risk-spreading strategy) for populations of these microorganisms under non-optimal growth conditions (15). In multicellular organisms, heterogeneity in the initial cell state can influence cell fate decisions, acting as an effective mechanism for development of higher-order structures such as the germ layers (16–19). Intriguingly, during cancer treatments, expression noise of drug-resistance genes can allow some cancer cells to cross survival thresholds, prompting difficulties in cancer therapy (20). Furthermore, preexisting or environment-induced noise can also contribute to the long-term evolutionary trajectory of microorganisms, as well as tumor progression, by increasing phenotypic diversification (21,22). Establishing how cells fine-tune expression noise could provide a better understanding of the regulatory mechanisms underlying population survival, adaptation during evolution, and cell development.

Previous studies have shown that noise largely originates from transcription bursts, in which short intervals of mRNA transcription are interspersed by long periods of negligible output (23,24). Such transcriptional fluctuations can be regulated by various cellular mechanisms (5). For instance, Rpd3(L), a well-known histone deacetylase complex involved in transcriptional repression, acts to reduce the frequency of transcriptional initiation and bursts, thereby resulting in increased noise levels (25). The noise arising from transcriptional bursts can be further amplified during mRNA processing and translation, leading to super-Poissonian variability in protein abundance (26). Moreover, phenotypic variation can be modulated by post-translational mechanisms, such as by the Hsp90 chaperone that regulates non-genetic variation by adjusting protein stability (27). Interestingly, gene expression noise in cells can vary in response to a fluctuating environment. For example, the methyltransferase Hmt1 functions as a hub of noise regulation by modulating nucleosome occupancy (14,28). When a population encounters environmental stress, *HMT1* expression is repressed, leading to increased cell-to-cell variation and ultimately enhanced population survival (14).

Translation processes may amplify and propagate mRNA fluctuations to the protein level (26). For instance, translational efficiency influences phenotypic noise in bacteria (29). Modulating the activity of the translation initiation factor eIF4G1 enables yeast cells to suppress global translation and increase protein expression noise (30). However, it remains unclear how general translational regulation affects cell-to-cell heterogeneity and which factors are involved.

In this study, we investigated regulators that can modulate expression noise. We established a dual-fluorescent reporter system in budding yeast and used experimental evolution to enrich for expression noise regulator mutants. We characterized the top identified regulator in depth, the histone deacetylase Hos2. Our results reveal that Hos2 regulates protein expression noise by adjusting the translation machinery, with *hos2* mutant cells displaying enhanced competitiveness in a frequently fluctuating environment. Our findings shed light on the relationship between noise regulation and translation status, representing a less well-studied topic compared to noise regulation at the transcriptional level.

## Materials and methods

### Yeast strains and genetic procedures

All yeast strains used in this study (Supplementary Table S1) were derived from the *S. cerevisiae* W303 strain (*leu2-3, 112 trp1-1 can1-100 ura3-1 ade2-1 his3-11, 15*). We used polymerase chain reaction (PCR) to amplify DNA fragments where necessary, and PCR products were purified by using an *AccuPrep* PCR/Gel purification kit (K-3037, Bioneer, USA). Yeast transformation was conducted by electroporation under 1,800 V/200 Ω/25 μF (BTX Gemini SC^2^, Harvard Bioscience, USA). After yeast transformation, strains in which final products had been correctly integrated were selected under a selection plate and checked by PCR and DNA sequencing.

To generate fluorescent reporters for measuring protein expression noise, we constructed reporter genes with their native promoters fused to fluorescent proteins at the 3’ end of the coding region followed by an *ADH1* terminator and selection markers. Reporter genes with a GFP tag and a *HIS3MX6* selection marker were directly amplified from the genomic DNA of corresponding strains in the yeast GFP collection (31). The amplified DNA fragments were assembled with the upstream and downstream regions of the *HIS3* locus into pRS41H plasmid by means of an In-Fusion HD Cloning Kit (#638933, Takara Bio USA Inc., USA). The mCherry tag and the selection marker *CaURA3* were amplified from the pFA6a-link-yomCherry-CaURA3 plasmid (32). The amplified DNA fragments were assembled with the reporter genes plus their native promoters, as well as the upstream and downstream regions of the *URA3* locus, into pRS41H plasmid by means of an In-Fusion HD Cloning Kit (#638933, Takara Bio USA Inc., USA). The assembled GFP- and mCherry-containing cassettes were further amplified by PCR and incorporated into the *HIS3* and *URA3* locus by replacing the coding region via homologous recombination, respectively. After yeast transformation, cells were spread on 2x complete supplement medium (2x CSM; 0.7% yeast nitrogen base without amino acids, 2% dextrose, 0.2% CSM mix powder; adjusted to pH 6) plates without histidine or uracil.

Gene deletion was performed by replacing the coding region with the KanMX4 cassettes amplified from the genomic DNA of corresponding strains in the yeast deletion collection (33) via homologous recombination. Cells were selected on rich medium (YPD; 1% yeast extract, 2% bactopeptone, and 2% dextrose) plates with 200 μg/ml G418.

All single nucleotide polymorphism (SNP) mutant reconstitution strains were constructed using the CRISPR/Cas9 MAGESTIC system (34). In brief, host cells were first transformed with the Cas9 expression plasmid containing the drug selection marker hphMX6 (Supplementary Table S2). The DNA sequences ± 80 basepairs from the desired SNP nucleotide were used to search for optimal guide RNA (gRNA) sequences using the online tool CRISpy-pop (https://crispy-pop.glbrc.org/). The donor DNA with the desired SNPs was amplified from the genomic DNA of evolved strains, and a heterology block was generated by PCR to disrupt the Cas9 target site in the donor DNA (35). These fragments were assembled with the gRNA-leading plasmid containing the *TRP1* selection marker using an In-Fusion HD Cloning Kit (#638933, Takara Bio USA Inc., USA). The assembled plasmid was then transformed into cells carrying the Cas9 expression plasmid, and the transformants were selected on 2x CSM plates without tryptophan and supplied with 300 μg/ml hygromycin B (HGB). The desired SNPs in the reconstitution strains were checked by DNA sequencing. To remove the plasmid, correct clones were grown in YPD overnight and plated on YPD plates. Colonies on the plates were replicated on YPD + HGB plates and then on 2x CSM tryptophan drop-out plates. Clones that did not grow on both plates were used for further experiments.

### EMS mutagenesis

To increase the genetic diversity of evolving populations, we performed EMS mutagenesis once before experimental evolution. Mutagenesis was performed according to a previously published protocol with some modifications (36). In brief, 5 × 10^6^ cells were washed once with sterile water and subsequently with 100 μl of phosphate buffer (0.1 M Na_2_HPO_4_ [pH 7.0]). The cells were then resuspended in 100 μl 4.2% EMS-containing phosphate buffer and maintained under constant shaking for 30 minutes at room temperature. To stop the reaction, 130 μl of 20% Na_2_S_2_O_3_ solution was added and the cells were then washed with sterile water. To constrain the population of mitochondria-defective cells that ubiquitously arise upon EMS treatment, 2 × 10^5^ cells were resuspended in 5 ml non-fermentable medium (YPG; 1% yeast extract, 2% bactopeptone, and 2% glycerol) for recovery for 18 hours before sorting. The survival rate of wild-type (WT) cells after EMS treatment ranged from 60% to 80%.

### Isolating clones that display increased protein expression noise following experimental evolution

After EMS mutagenesis, we used fluorescence-activated cell sorting (FACS) to enrich for clones that exhibited increased protein expression noise. Cell sorting was conducted in a BD FACSJazz machine (BD Bioscience, USA) at a flow rate of ∼3000 events/sec. The GFP and mCherry fluorescent reporters were excited by 488 nm and 561 nm lasers and readouts were acquired using 513/17 nm and 610/20 nm bandpass filters, respectively. To eliminate interference from small particles in the fluidic system, we excluded signals with forward scatter (FSC) <2570 as a trigger threshold. We used two hierarchal gates to gate the population for sorting. First, using the FSC-Width vs FSC-Area distribution, we gated out cells with higher FSC-Width, which corresponds to cell doublets or aggregates. Second, using the FSC-Area vs side scatter (SSC-Area) distribution, we gated for cells displaying a uniform physiology by contour gating 80% of the population. The dual-gated populations were used for further cell sorting.

During each sorting event, only 10% of the outermost cells of the population were collected because cells in which protein expression noise regulators have been disrupted by mutagenesis are more likely to be localized in extreme regions of the distribution. For each sorting event, a total of 1 × 10^5^ cells were sorted and recovered for 48 h in 3 ml YPG at 28°C to restore the population size. The cells were then diluted 500-fold and refreshed in 3 ml YPG at 28°C for 18 h before the next sorting event. To prevent bacterial contamination from the sorter, chloramphenicol (125 μg/ml) and doxycycline (125 μg/ml) were added to the recovery medium. Sorting criteria are described in the Results section.

### Quantitating protein expression noise by flow cytometry

To measure expression noise levels, cells from solid plates were inoculated in 3 ml YPD and grown at 28°C for 18 h. The cell density of the overnight culture was measured using a spectrophotometer. All protein expression noise levels were measured under respiratory growth conditions (YPG), except for in Figure 4A. To measure protein expression noise in the glycerol-based growth condition (YPG), 9 × 10^4^ cells from overnight culture were propagated in 3 ml YPG and grown at 28°C. For Figure 4A, 1.2 × 10^4^ cells from overnight culture were propagated in 3 ml YPD with or without the indicated concentrations of cycloheximide and grown at 28°C. Next, we collected 0.6 OD_600_ cells from the early-log phase (0.3–0.5 OD_600_/ml) culture and resuspended them in 1 ml filter-sterilized phosphate-buffered saline (PBS) (137 mM NaCl, 2.7 mM KCl, 10 mM Na_2_HPO_4_ and 1.8 mM KH_2_PO_4_ [pH 7.0]). Using a BD FACSymphony™ A3 Cell Analyzer (BD Bioscience, USA), we measured the fluorescence intensity of GFP and mCherry at a flow rate of ∼3000 events/s. We used the same gating criteria as described above for cell sorting to eliminate machine noise, particles in the fluidic system and cell doublets to gate the populations for data recording. Signals from 30 000 cells were recorded for each sample.

We used FlowJo™ v10 Software (BD Biosciences, USA) for data analysis. According to the FSC-A versus SSC-A distribution, we gated for cells displaying uniform physiology by contour gating 80% of the population. Fluorescence readouts of the entire population were used to calculate protein expression noise after log-transformation. Protein expression noise is calculated as the degree of variation of the fluorescence reporters. We used the Fano factor, which is the ratio of variance over the mean (σ^2^/μ), to represent protein expression noise levels. This factor is characterized by having less interference from changes in protein abundance and it is more sensitive to variation-driven increased noise (29). To measure the effects of Hos2 on intrinsic and extrinsic noise, we used the WT and *hos2*Δ strains harboring Tdh2-GFP and Tdh2-mCherry reporters. The values of both reporters from every individual cell were extracted, and the intrinsic noise (η_int_), extrinsic noise (η_ext_) and total noise (η_tot_) were calculated as previously described (1).

### Whole-genome sequencing analysis

Genomic DNA from 5 OD_600_ yeast cells grown in YPD medium overnight was extracted according to a phenol–chloroform method, as described previously (14), and quantified using a ND-1000 spectrophotometer (Nanodrop Technology, USA). The extracted genomic DNA treated with RNase A and precipitated by absolute ethanol was further purified by means of a Genomic DNA Clean & Concentrator-25 kit (D4065, Zymo Research). Concentrations of the purified genomic DNA were determined using a Qubit dsDNA BR assay kit (Thermo Fisher Scientific).

A Truseq DNA PCR-free LT kit (Illumina, USA) was used for library preparation and a NextSeq Mid Output 500 system (Illumina, USA) was employed for DNA sequencing. At least 100-fold coverage was achieved for the sequencing of evolved clones and it was 200-fold for pooled ancestral clones and pooled segregants. Illumina bcl2fastq2.17 was used to generate fastq files and for demultiplexing. The raw reads were trimmed in trimommatic v.0.36 (37). Genomic variants were identified according to the following pipeline: raw reads were mapped to the W303 reference genome (38) by BWA-MEM (39); GATK4 (Van der Auwera & O’Connor, 2020) was used for variant calling; and SnpEFF (40) was used to annotate and predict variant effects. We used R for data trimming and variant reporting. Only variants with coverage > 30 and an allele frequency > 0.5 were considered.

### Bulk segregant analysis

We performed bulk segregant analysis to identify the causal mutations in evolved clones. The evolved lines were mated with the ancestral lines. The obtained diploid cells were grown in sporulating media (0.1% yeast extract, 0.05% dextrose and 2% potassium acetate; adjusted to pH 9) for 3–5 days to induce sporulation. The cells (20 μl) were then harvested and treated with 0.5 mg/ml Zymolyase-100T (Nacalai Tesque, Japan) at 28°C for 15–30 min to digest cell walls. The suspension was streaked across a YPD plate and at least 16 tetrads were dissected per strain. A total of three replicates of protein expression noise measurement were conducted to classify progenies into ‘ancestral-like’ or ‘evolved-like’. For each bulk, 20 progenies exhibiting a consistent phenotype across the three replicates were pooled and subjected to whole genome sequencing analysis.

Equal amounts of cells were pooled and their genomic DNA was extracted. At least 200 × coverage was achieved for sequencing. We applied QTL-seq (41,42) to identify quantitative trait loci from two populations of segregated progeny. To filter out spurious SNPs caused by sequencing or alignment errors, only SNPs with an SNP index >0.3 in at least one bulk were considered. Mutations with a ΔSNP index (i.e. the evolved SNP-index subtracted by the ancestral SNP-index) of >0.8 were considered as causal mutations for increasing protein expression noise in the evolved clones.

### Total RNA extraction and DNase treatment

To collect yeast cells for total RNA extraction, 3 ml pre-cultures in YPD medium were generated by inoculating a single colony from a freshly streaked plate and incubating overnight at 28°C. The pre-cultures were diluted to 2.5 × 10^−3^ OD_600_/ml in a volume of 30 ml YPG medium and incubated at 28°C until early-log phase (0.3–0.5 OD_600_/ml). Approximately 10 OD_600_ cells were harvested by centrifugation at 4°C, washed once with 1 ml of ice-cold nuclease-free water (UltraPure™ DNase/RNase-Free Distilled Water, 10977015, Invitrogen, USA), and stored at −80°C.

Cells were thawed on ice and resuspended in 500 μl of ice-cold freshly prepared DEPC-treated lysis buffer (0.1 M Tris–HCl [pH 7.5], 0.1 M LiCl, 2% β-ME, 0.01 M EDTA [pH 8.0] and 5% SDS). The mixtures were immediately transferred to 2-ml breaking tubes (72.693, Sarstedt, Germany) containing ice-cold 500 μl PCIA (phenol:chloroform:isoamyl alcohol = 25:24:1 [pH 4.5]; 0.1% 8-hydroxyquinoline) and 0.3 g glass beads (11079105, BioSpec Products, USA). Cells were broken by means of a FastPrep-24™ 5G homogenizer (MP Biomedicals™, USA) at a speed of 6 m/s for 40 s at room temperature. After centrifugation (13 000 rpm for 10 min at 4°C), the aqueous phases were further extracted by two rounds of 200 μl ice-cold acidic PCIA treatment and the nucleic acids were precipitated adding 2.5 volumes of absolute ethanol for 2 h. RNA samples were resuspended in 200 μl nuclease-free water and quantified using an ND-1000 spectrophotometer (Nanodrop Technology, USA).

To remove DNA contaminants, 35 μg of total RNA was treated with a TURBO DNA-free™ kit (AM1907, Invitrogen, USA) in a 50-μl reaction. Final RNA quality was checked by 28S/18S quantification on an Agilent TapeStation 2200 system using RNA screen tape (Agilent Technologies, USA). The DNase-treated RNAs were stored at −80°C for subsequent RT-qPCR or RNA-seq analyses.

### Reverse transcription (RT) and quantitative-PCR (qPCR) analyses

The qPCR reactions were carried out in 96-well plates in a QuantStudioTM 12 K Flex Real-Time PCR System (Applied Biosystems by Thermo Fisher Scientific) in 20 μl volumes using Fast SYBR^TM^ Green Master Mix (4385612, Applied Biosystems by Thermo Fisher Scientific). *UBC6* transcripts were used as an endogenous control for qPCR. Average ΔΔ*C*_T_ and standard deviation values were determined from at least three technical repeats. The relative fold-change in expression for each gene is shown according to the 2^−ΔΔCT^ method.

### RNA-seq analysis

RNA-seq libraries were prepared with a TruSeq Stranded mRNA HT Sample Prep Kit (Illumina, USA) by using 2 μg of total RNA and sequenced for paired-end 150-bp read length according to the protocol of a NextSeq 500 High Output kit v2.5 (75 cycles) on an Illumina NextSeq 500 system. Raw reads were quality-trimmed using Trimmomatic v0.38 (37). The relative abundance of each gene in units of Transcripts Per Million (TPM) for each sample was quantified using Salmon v1.4.0 (43). Default settings in Tximport (44) were used to convert the estimated transcript abundance files from Salmon to a DESeq2-compatible dataset. DESeq2 (45) was used for the differential analysis of gene expression in R v4.0.4 with default settings. In DESeq2, the *P*-values attained by Wald test were corrected for multiple testing using the Benjamini and Hochberg method. Genes with mean TPM <1 in all conditions were omitted from analyses. Differences in gene expression with an adjusted *P*-value <0.05 were considered statistically significant.

### Gene Ontology (GO) analysis

Statistically significant differentially expressed genes (DEGs) in the *hos2* mutant were separated into two groups, i.e. down-regulated and up-regulated genes. GO analysis was performed using the online tool AmiGO2 (46). GO terms of biological process (BP), molecular function (MF) and cellular component (CC) were analyzed separately for both gene groups. Only GO terms with adjusted *P*-value <0.05 and with >1-fold enrichment score were considered further. For clarity, only the bottom layer GO terms in each cluster are shown.

### Total protein extraction and protein abundance measurement

Single colonies from freshly streaked plates were inoculated in 3 ml YPD medium and grown overnight at 28°C. The pre-cultures were diluted to 3.125 × 10^−4^ OD_600_/ml in 12 ml YPD medium or 2.5 × 10^−3^ OD_600_/ml in 12 ml YPG medium, and incubated at 28°C until early-log phase (0.3–0.5 OD_600_/ml). 3 OD_600_ cells were harvested by centrifugation at 4°C, washed once with 1 ml of ice-cold water, and stored at −80°C.

Cells were thawed on ice, resuspended in 1 ml of freshly prepared lysis buffer (0.24 M NaOH and 1% β-ME), and incubated on ice for 15 minutes. The mixtures were immediately mixed with 150 μl of 55% Trichloroacetic acid (TCA, T9159, Sigma-Aldrich, USA) and incubated on ice for 10 minutes to precipitate proteins. After centrifugation (13 000 rpm for 10 min at 4°C), the supernatants were discarded, and the precipitates were dissolved in 150 μl of HU sample buffer (8 M urea, 0.1% SDS, 0.2 M Tris–HCl [pH 6.5], 1 mM EDTA [pH 8.0], 120 mM Tris, and freshly added 5% β-ME) by incubating at 65°C for 40 min. The samples were stored at −80°C.

We used the Bradford protein assay to measure the total protein abundance. Samples stored at −80°C were thawed on ice and centrifugated (13 000 rpm for 1 min). The aqueous phases were diluted for 5 folds by water, and 10 μl of the diluted samples were transferred into a 96-well plate (Tissue Culture Testplate 96 wells F-bottom, 92096, Techno Plastic Products, Switzerland). The 5-fold diluted HU sample buffer was used as a blank. Subsequently, 200 μl of 5-fold diluted Protein Assay dye Reagent Concentrate (#5000006, Bio-Rad, USA) was added into each well. The mixtures were incubated on the shaker at room temperature for 10 min, and analyzed using a Tecan plate reader (Infinite 200 PRO, Tecan, Switzerland) with OD_595_ absorbance. The protein concentrations were calculated by a standard curve generated via bovine serum albumin (BSA).

### Polysome profiling

To conduct polysome profiling, 3 ml pre-cultures in YPD medium were generated by inoculating a single colony from a freshly streaked plate and incubating overnight at 28°C. Approximately 1.2 × 10^6^ cells from the overnight culture were propagated in 40 ml of YPG medium and grown to early-log phase (0.3–0.5 OD_600_/ml) at 28°C. Yeast cultures were treated with 50 μg/ml cycloheximide (CHX) for 15 min at 28°C before harvesting by centrifugation (4000 rpm for 5 min at 4°C). Pelleted cells were washed with ice-cold lysis buffer (20 mM Tris–HCl [pH 7.5], 8 mM MgCl_2_ and 100 mM KCl) and stored at –80°C before further extraction.

Cell pellets were resuspended in 400 μl ice-cold lysis buffer with freshly added 12 mM β-mercaptoethanol (β-ME), 200 μg/ml CHX and 1 mM PMSF, together with a half-volume of 0.5 mm glass beads. Cells were disrupted by vortexing for 30 seconds and then cooling down on ice for 1 minute, for a total of six cycles. Unbroken cells, large debris, and glass beads were removed by centrifugation (13 000 rpm for 10 min at 4°C). For polysome profiling, 9 OD_260_ units of yeast cell extracts were layered onto a 12 ml linear sucrose gradient (7–47%) prepared in lysis buffer with freshly added 12 mM β-ME and 200 μg/ml CHX. These gradients were centrifuged in an SW-40 rotor (Beckman Coulter, USA) at 36,000 rpm for 2.5 h at 4°C. The fractions were analyzed using a BR-188 Density Gradient Fractionation System (Brandel, USA) with *A*_254_ absorbance. Polysome to monosome (P/M) ratios were calculated by comparing the combined area under the polysome peaks and the 80S peaks.

### Growth rate measurement

To measure growth rates, 3 ml pre-cultures in YPD medium were generated by inoculating a single colony from a freshly streaked plate and incubating overnight at 28°C. Approximately 9 × 10^4^ cells from the overnight culture were propagated in 3 ml of YPG medium and grown to early-log phase (0.3–0.5 OD_600_/ml) at 28°C. The refreshed cell cultures were diluted to a concentration of ∼1.5 × 10^5^ cells/ml in fresh YPG with or without translation inhibitors. Then, 120 μl of the diluted culture from each sample was transferred into a 96-well plate (Tissue Culture Testplate 96 wells F-bottom, 92096, Techno Plastic Products, Switzerland). Cell growth was measured at OD_595_ and ‘2 × 2 multiple reads per well’ mode every 12 min using a Tecan plate reader (Infinite 200 PRO, Tecan, Switzerland) at 28°C. Each 12-min cycle included 3 min of reading, 1 min of shaking, 3 min of standing, 1 min of shaking, 3 min of standing and 1 min of shaking. Tecan software Magellan Version 7.2 was used for data acquisition and analyses. Maximal growth rates were determined by calculating the maximum slope of each growth curve based on 20 time points in Magellan software.

### Cell growth under a fluctuating environment

Relative fitness was measured by means of competition assay. In the experimental set, the *hos2*Δ strain with fluorescent reporters (and selectable marker genes *HIS3* and *URA3*) was competed against the WT strain (W303) without fluorescent signal. A control set of cultures were also established, in which the ancestral wild-type strain with the same fluorescent reporters was competed against the WT strain without the reporters. Overnight WT and *hos2*Δ (or reporter-carrying ancestral) cultures were refreshed to early-log phase in YPG and mixed in a 1:1 ratio based on cell numbers. The proportion of the fluorescence-positive population in the mixtures was measured by flow cytometry. Mixtures were then inoculated into YPG or YPG + 0.25 M NaCl until early-log phase (OD_600_ 0.3–0.5). The cells in YPG were consistently refreshed with YPG when the culture reached early-log phase during the experimental period. The cells in YPG + 0.25 M NaCl were separated into two groups. One group was continuously refreshed in YPG + 0.25 M NaCl when the culture reached early-log phase, which was defined as the constant condition. The other group was refreshed in alternating conditions of YPG or YPG + 0.25 M NaCl, which was defined as the fluctuating growth condition. Proportions of fluorescent-positive populations for all three conditions were measured periodically and the ratios were calculated. The *hos2*Δ-to-WT ratio was always normalized to the ancestral-to-WT ratio in the same condition to control the marker gene effect.

### Statistical analysis

Details of statistical analyses are presented in the main text or corresponding figure legends. Statistical significance was established using an unpaired Student's *t*-test or a Mann–Whitney *U* test with suitable correction in GraphPad Prism version 10.0.2 for Windows (GraphPad Software, Boston, Massachusetts USA) or the default functions packaged in each analysis tool.

## Results

### A dual-reporter experimental evolution strategy for obtaining high-noise mutants

**Figure 1.**
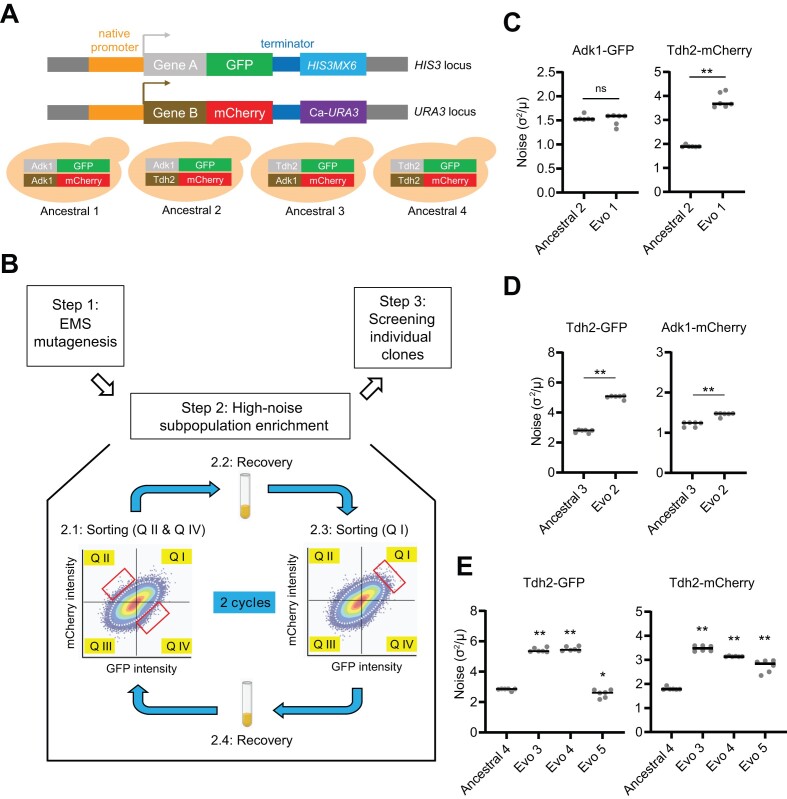
Experimental evolution generates five clones displaying noisy protein expression. (**A**) Schematic of the dual-reporter system for quantifying protein expression noise and the ancestral clones used for conducting experimental evolution (see Materials and methods for details). (**B**) Schematic of the experimental evolution experiment. In Steps 2.1 and 2.3, signals outside the dotted white circle indicate the 10% outermost cells based on the signal intensity of both reporters. The red boxes indicate the selected subpopulations at each step. In Steps 2.2 and 2.4, selected cells were grown in YPG to recover the population size. See Materials and Methods for details. EMS, Ethylmethanesulfonate; Q, quadrant. Created with BioRender.com. (C–E) Expression noise levels of the evolved clones from the ancestral 2 (**C**), ancestral 3 (**D**), and ancestral 4 strains (**E**). The fluorescence signals of both reporters in each clone were measured by flow cytometry and the Fano factor (calculated as a ratio of variance to mean, σ^2^/μ) was used to quantify protein expression noise. Horizontal solid lines represent median values for replicates. *P< 0.05, **P< 0.01 (two-sided Mann–Whitney *U* test, *n* = 6). See Supplementary Table S6 for raw data and the details of statistical analysis. See also Supplementary Figure S1 for the relative expression noise levels of sorted subpopulations.

Alternating selection between two subpopulations with extreme expression levels of fluorescent reporters using a fluorescence-activated cell sorter (FACS) can efficiently enrich for bacterial and yeast cell clones displaying elevated protein expression noise (14,47). Our previous study showed that half of the evolved lines carrying different reporters exhibited significantly increased expression noise levels after 35 cycles of mutagenesis and cell sorting (14). However, such a selective regime was time-consuming, and the evolved cells often accumulated hundreds to thousands of mutations after many cycles of selection. To better identify the genetic basis of noise regulation, a more efficient strategy is required. In this study, we aimed to identify general regulators of expression noise in a less tedious way using a reduced number of mutagenesis cycles to save time and avoid accumulating too many mutations in the evolved clones. We established a dual-reporter system by fusing one reporter gene to a green fluorescent protein (GFP) and the other to a red fluorescent protein (mCherry), and drove their expression via the respective endogenous promoters (Figure 1A), which allowed us to quantify protein expression noise. To increase the likelihood of identifying noise regulators functioning in multiple pathways, we selected *TDH2* and *ADK1* as our reporter genes. *TDH2* encodes a glyceraldehyde-3-phosphate dehydrogenase (GAPDH) involved in glycolysis and gluconeogenesis (48), whereas *ADK1* encodes an adenylate kinase involved in purine metabolism (49). Four strains each harboring two fluorescent reporters were created as the ancestral lines for further experimental evolution (Figure 1A).

We anticipated that the protein expression noise of the reporter genes would change if the function of a noise regulator became disrupted. To increase the genetic diversity of evolving populations, the ancestral yeast cells were treated once with a mutagen (4.2% ethyl methanesulfonate; EMS) before selection (Figure 1B). To enrich for subpopulations displaying extreme expression levels in both reporters, only the 10% outermost cells were collected at each enrichment step. At the first step of selection, the subpopulations displaying high GFP but low mCherry signals (Figure 1B, gate Q IV) and those possessing high mCherry but low GFP signals (Figure 1B, gate Q II) were sorted (see Materials and methods for details). Doing so reduced the possibility of obtaining cells carrying mutations in general activators or repressors of gene expression. To prevent isolating cells with mitochondrial defects, the sorted cells were grown in non-fermentable media (Yeast extract-Peptone-Glycerol; YPG) to restore the population size. After recovery, the subpopulations exhibiting high GFP and mCherry expression (Figure 1B, gate Q I) were sorted to avoid obtaining cells with defective reporter genes. Protein expression noise levels of the sorted subpopulations were first recorded at this step (Step 2.3 in Figure 1B), and the cell populations were allowed to recover again in YPG for the second round of selection. After two rounds of selection, we observed that the variability of reporter protein abundance among cells had increased (Supplementary Figure S1A), which may come from genetic or nongenetic changes of the cells in the population. We examined individual clones from each evolved population to screen for those showing increased protein expression noise. Ultimately, we obtained five evolved clones (Evo1–Evo5) manifesting significantly increased protein expression noise after experimental evolution (Figures 1C–E). We isolated high-noise clones from three of the four evolved populations, i.e. except from the one carrying both fluorescent proteins fused with *ADK1* (Supplementary Figure S1B). We also noticed that Evo5 displayed inconsistency between the two *TDH2* reporters but, in any case, we could not analyze this clone further since it lacked the ability to mate (see the next section).

To investigate the genetic basis of the increased noise in our evolved clones, we subjected both ancestral and evolved cells to whole-genome sequencing, which revealed 38, 48, 38, 34 and 54 mutations in the genomes of the five evolved clones (Supplementary Figure S1C and Supplementary Table S3). Thus, compared to our previous selection regime that generated 1022 mutations in the evolved clone (14), the system reported herein proved more efficient. Reducing the numbers of mutations in evolved clones in this way should decrease the likelihood of suppressive effects among mutations and allow us to more easily pinpoint causal variants. Thus, we have developed an improved strategy to collect cell lines displaying increased expression noise using experimental evolution, which provides a useful approach for studying non-genetic variation.

### Mutations in Hos2 and Snf2 contribute to increased expression noise in our evolved clones

**Figure 2.**
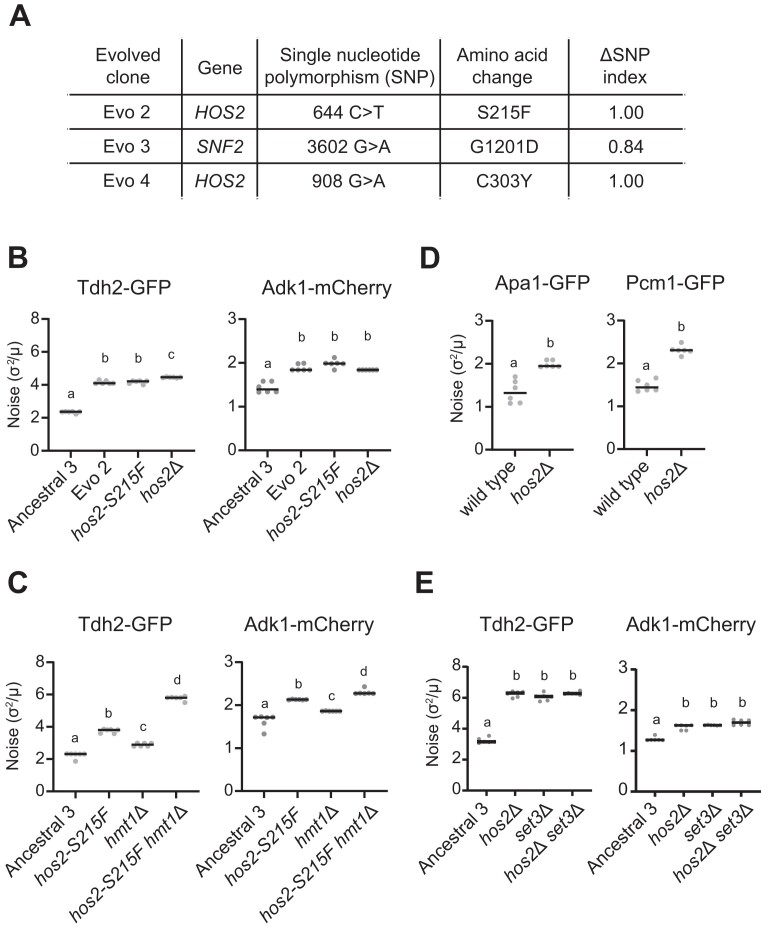
The histone deacetylase Hos2 acts as a noise regulator. (**A**) Candidate causal mutations leading to increased expression noise in the evolved clones. The ΔSNP index was calculated as the frequency difference between evolved-like and ancestral-like F1 segregants. See also Supplementary Figure S2. (**B**) *hos2-S215F* and *hos2Δ* mutant cells exhibit increased expression noise levels compared to ancestral cells, but similar levels to the Evo 2 clone (Kruskal–Wallis test, *P* = 2.12 × 10^–4^ for Tdh2-GFP and *P* = 3.624 × 10^–4^ for Adk1-mCherry, *n* = 6). The *hos2-S215F* mutation was reconstituted in the ancestral strain by CRISPR/Cas9. (**C**) Deleting *HMT1* in the *hos2-S215F* mutant further increases the protein expression noise of both reporters, suggesting an additive effect on noise regulation (Kruskal–Wallis test, *P* = 7.517 × 10^–5^ for Tdh2-GFP and *P* = 6.859 × 10^–5^ for Adk1-mCherry, *n* = 6). (**D**) *hos2Δ* cells display increased expression noise for Apa1-GFP and Pcm1-GFP, indicating that Hos2’s role in noise regulation is not specific to one or two pathways. (**E**) Deleting *SET3* increases expression noise levels to those of *hos2Δ* cells, while the *hos2Δ set3Δ* double mutant does not show an additive effect on noise (Kruskal–Wallis test, *P* = 1.816 × 10^–3^ for Tdh2-GFP and *P* = 4.015 × 10^–4^ for Adk1-mCherry, *n* = 6). In (B–E), a two-sided Mann–Whitney *U* test with Bonferroni correction was used for multiple comparisons, with different letters above each column indicating statistically significant differences (adjusted *P*-value < 0.05). Horizontal solid lines represent median values for replicates. Supplementary Table S6 for raw data and the details of statistical analysis.

To identify the causal mutation that led to increased expression noise in the evolved clones, we performed bulk segregant analysis by crossing the evolved cells with their ancestral cells and analyzing their F1 haploid progeny. Unfortunately, Evo1 and Evo5 had lost the ability to mate, likely due to mutations in their respective genomes that abolished their mating capability. In Evo1, we found a mutation (C513Y) in the NAD^+^-dependent histone deacetylase-coding gene *SIR2* (Supplementary Table S3), which has been shown that the null mutant diminishes the mating pheromone response (50). A mutation (D95N) in the functional unknown gene *ABM1* and a mutation (P304L) in the DNA endonuclease gene *SLX4* were observed in Evo5 (Supplementary Table S3). A genome-wide screen study indicated that deleting the *ABM1* or *SLX4* gene decreases silencing at the silent mating type locus, which may lead to defects in the mating ability (51). Therefore, we could only analyze Evo2, Evo3 and Evo4 further. We measured protein expression noise levels of both reporter genes in 64 F1 segregants and established an ‘ancestral-like’ pool and an ‘evolved-like’ pool from each evolved clone. Twenty segregants with consistent phenotypes from both pools were then selected for whole-genome sequencing (Supplementary Figure S2). The ΔSNP index was then determined (evolved SNP index subtracted by the ancestral SNP index, also see Materials and methods) (42). In Evo2 and Evo4, we observed mutations (S215F and C303Y) in the histone deacetylase-coding gene *HOS2* only in the evolved-like pool (ΔSNP index = 1) (Figure 2A and Supplementary Table S3). In Evo3, we also uncovered a mutation (G1201D) in the chromatin remodeler catalytic subunit-coding gene *SNF2* primarily in the evolved-like pool (ΔSNP index = 0.84) (Figure 2A and Supplementary Table S3). These findings indicate that both *HOS2* and *SNF2* are involved in noise regulation (Figure 2A and Supplementary Table S3).

To confirm that the identified genes contribute to noise regulation, we introduced the evolved SNPs into the ancestral lines by means of the CRISPR/Cas9 system. The *hos2-S215F* mutant showed similar expression noise levels to evolved clone Evo2 in both the Tdh2-GFP and Adk1-mCherry reporters, indicating that *hos2-S215F* is the primary mutation causing enhanced expression noise in the evolved clone (Figure 2B). The S215 residue in *HOS2* is highly conserved across species (Supplementary Figure S3A), implying that this residue is crucial to protein function or structure. Indeed, *hos2*Δ cells also display increased expression noise, supporting that the increased noise level in the evolved clone is caused by Hos2 loss-of-function (Figure 2B).

Snf2 is a catalytic subunit of the SWI/SNF chromatin-remodeling complex (52). The reconstituted *snf2-G1201D* mutant exhibited increased noise in the Tdh2 reporter, but its noise level was significantly lower than that of the Evo3 evolved clone (Supplementary Figure S3B). Therefore, other mutations may exist that also contribute to the increased noise observed for Evo3. Snf2 is a methylation substrate of the methyltransferase Hmt1 (53). Our previous study revealed that the function of SWI/SNF complexes is compromised in the *hmt1* mutant (14). Moreover, there was no significant additive effect on noise levels for the *snf2-G1201D hmt1*Δ double mutant (Supplementary Figure S3C), indicating that these two genes operate in a similar pathway. In contrast, the *hos2-S215F hmt1*Δ double mutant exhibited a higher noise level than the single mutants (Figure 2C), raising the possibility that Hos2 may be a novel regulator that modulates protein expression noise independently of Hmt1. Therefore, we decided to focus on the noise regulation mechanism controlled by Hos2 for subsequent experiments.

### The Set3/Hos2 deacetylase complex acts as a general noise regulator

The overall expression noise within an identical population of cells is usually described as the sum of two types of expression stochasticity, which are referred to as intrinsic and extrinsic noise. Intrinsic noise (denoted η_int_) reflects the discrete nature of biochemical events inherent to the gene expression process, such as the times and orders of transcription and translation. Extrinsic noise (denoted η_ext_) arises from the fluctuations of intra- and extracellular environments, such as the concentration of RNA polymerases and ribosomes, the stage in the cell cycle, and the micro-environment of cells (1,54). To investigate the effects of Hos2 on different types of noise, we used the strains harboring dual-reporters of the same gene (i.e. Tdh2-GFP and Tdh2-mCherry) (1). Both intrinsic and extrinsic noise increased in the *hos2*Δ mutant (Supplementary Figures S3D and S3E), suggesting that Hos2 is involved in different layers of noise regulation, and may act as a global regulator of expression noise.

To ensure that Hos2 works as a general regulator of expression noise for multiple pathways, we tested the effect of *HOS2* deletion (*hos2*Δ mutant) on another two reporter genes—*APA1* and *PCM1*—which are involved in pathways distinct from those of *TDH2* and *ADK1*. *APA1* encodes an Ap4A phosphorylase that functions in nucleotide biosynthesis (55), whereas *PCM1* encodes an N-acetylglucosamine-phosphate mutase required for chitin biosynthesis (56,57). We found that the protein expression noise levels of both selected reporter genes were increased in the *hos2*Δ mutant, highlighting that the regulatory function exerted by Hos2 is not specific to one or two pathways (Figure 2D).

The histone deacetylase Hos2 is a subunit of the Set3 deacetylase complex (58,59). To establish if the effect of Hos2 is mediated through the Set3 deacetylase complex, we measured expression noise in a *SET3* deletion mutant (*set3Δ*), a gene encoding the major component of the Set3 complex (58). Expression noise levels of *set3Δ* cells were similar to those of *hos2*Δ and *hos2*Δ *set3Δ* cells, supporting that the Set3/Hos2 histone deacetylase complex is involved in noise regulation (Figure 2E).

### Hos2 is involved in regulating translation

Expression noise is coupled to cell growth. Noise is generally higher in cells with lower growth rates (60). Under the YPG condition, the growth kinetic curves of WT and *hos2*Δ cells were similar in the early-log phase (Supplementary Figure S3F), and deleting *hos2* only caused a slight reduction in the maximum growth rate (Supplementary Figure S3G). These results suggest that slow growth is not the major cause of increased noise in the *hos2*Δ mutant.

**Figure 3.**
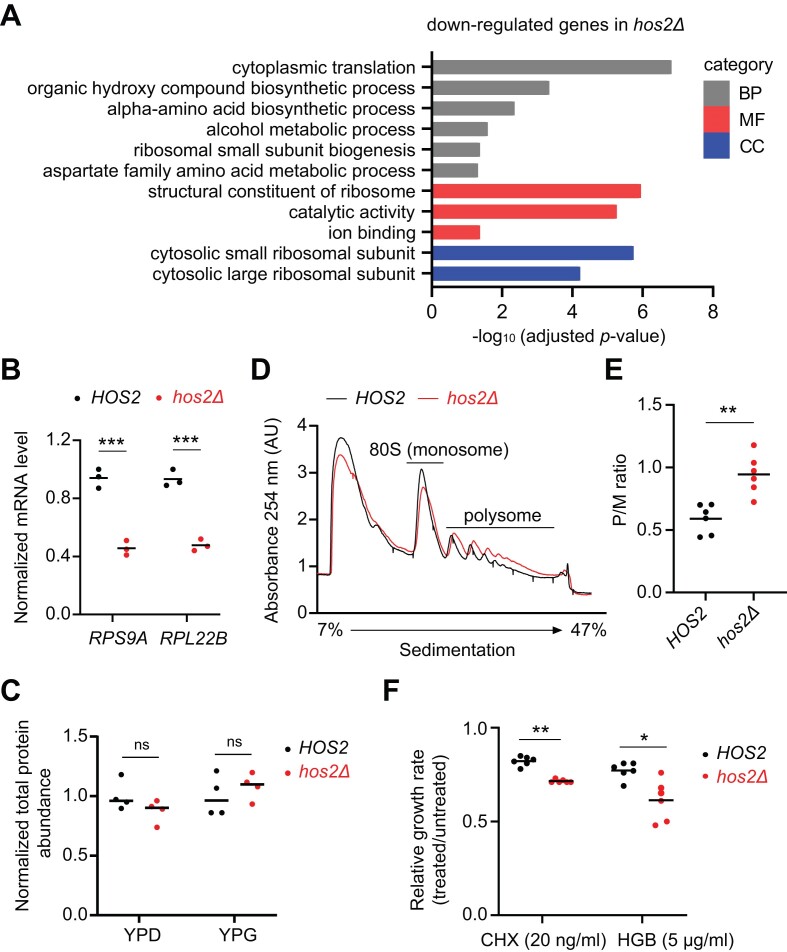
Hos2 is involved in translational regulation. (**A**) Gene ontology (GO) analysis of down-regulated genes in the *hos2*Δ mutant. The x-axis represents the –log_10_-adjusted *p*-values for distinct GO terms. Only GO terms displaying >1-fold enrichment scores are considered. BP, biological process; MF, molecular function; CC, cellular component. See Supplementary Table S4 for the complete list of DEGs and Supplementary Table S5 for details of enriched GO terms in the DEGs of the *hos2*Δ mutant. (**B**) RT-qPCR validation of ribosomal protein gene down-regulation in *hos2*Δ cells. *UBC6* was used as the internal control. The relative expression levels were calculated by normalizing the *hos2*Δ values against WT values. ***P-value < 0.001 (two-sided unpaired *t*-test with Welch's correction, *n* = 3). (**C**) The WT and *hos2Δ* cells have similar amounts of total protein in both YPD and YPG conditions. The relative protein levels were calculated by normalizing against the average values of WT in each condition, respectively. ns, no significant difference (two-sided Mann–Whitney *U* test, *n* = 4). (**D**) Polysome profiling of WT and *hos2*Δ mutant strains. Individual polysome profiles were measured by tracing the UV absorbance (*A*_254_) after fractionating the whole-cell extract using a 7–47% sucrose density gradient. The polysome to monosome (P/M) ratio shown in (**E**) was calculated from the area under the curve. (**E**) *hos2*Δ mutant cells have a higher P/M ratio than WT cells. **P-value < 0.01 (two-sided Mann–Whitney *U* test, *n* = 6). (**F**) *hos2*Δ mutant cells are more sensitive to translation inhibitors than WT cells. The relative growth rate was calculated as the ratio of the maximum growth rates between drug-treated and untreated cells. CHX: cycloheximide. HGB: hygromycin. *P-value < 0.05; **P-value < 0.01 (two-sided Mann–Whitney *U* test, *n* = 6). Horizontal solid lines represent median values of replicates. See Supplementary Table S6 for raw data and the details of statistical analysis.

Hos2 may directly regulate the expression noise of reporter genes by modifying nucleosomes near their promoters (28). We examined the data from a previous chromatin immunoprecipitation (ChIP)-on-chip experiment (61) and found that our reporter genes are not Hos2 targets, implying an indirect effect. To further dissect the regulatory mechanism of Hos2 in protein expression noise, we performed RNA sequencing (RNA-seq) and compared the transcriptomes between wild-type (WT) and *hos2*Δ mutant cells grown in YPG. Although Hos2 was initially identified as a meiotic-specific repressor of sporulation genes, it also functions as a transcription activator by deacetylating histones in the coding region, thereby reverting chromatin to the original permissive state (58,59). In our transcriptomic analysis, we identified 1156 differentially expressed genes (DEGs) that exhibited significant differences between the *hos2*Δ and WT cells (Supplementary Table S4). Gene ontology (GO) analysis on the 598 down-regulated genes in *hos2*Δ cells revealed enrichment in multiple categories related to translation (Figure 3A and Supplementary Table S5). We measured mRNA expression of two selected ribosomal protein genes by real-time qPCR to further validate that Hos2 is required for the expression of certain ribosomal protein genes (Figure 3B). Consistent with our transcriptomic data, the previous ChIP-on-chip analysis also showed that Hos2 preferentially affects histone acetylation in the coding region of ribosomal protein genes (61).

To better understand the effect of Hos2 on translation, we examined the total protein abundance in early-log phase cells. No significant difference was observed between the *hos2*Δ and WT cells (Figure 3C), indicating that the *hos2* mutation does not have a severe effect on global protein abundance. However, when we performed polysome profiling by sucrose gradient ultracentrifugation, it revealed an elevated accumulation of ribosomes on mRNAs in the *hos2*Δ mutant relative to WT (Figure 3D) and that the polysome-to-monosome (P/M) ratio was increased ∼1.5-fold in the mutant compared to WT (Figure 3E). Mutations affecting translational elongation or termination have been shown to increase the P/M ratio (62,63). To further examine if disrupting *HOS2* altered the translation machinery, we treated WT and *hos2*Δ mutant cells with a mild dose of one of two translation inhibitors, cycloheximide (CHX) or hygromycin B (HGB). We anticipated that these treatments would only slightly inhibit growth of WT cells but elicit more severe effects in the mutant cells in which translation is partially compromised. Indeed, the *hos2*Δ mutant line presented significantly lower growth rates compared to WT under the CHX and HGB treatments (Figure 3F). Together, these results indicate that the protein translation machinery is partially compromised in the *hos2*Δ mutant.

### Translation inhibition enhances protein expression noise levels

**Figure 4.**
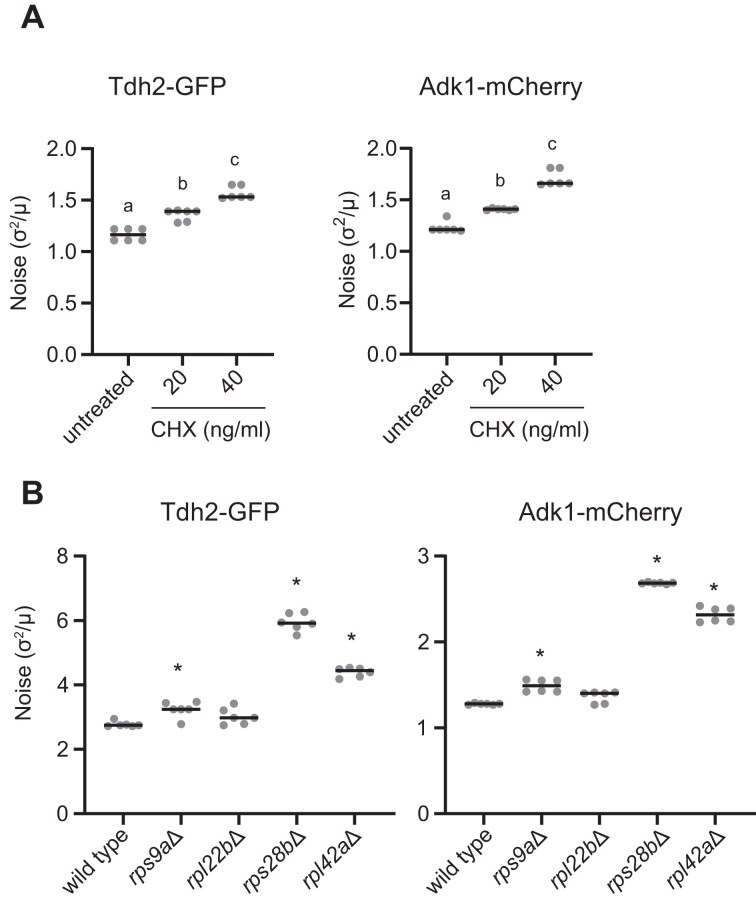
Compromising the translational machinery increases noise levels. (**A**) Expression noise levels are increased in WT cells treated with mild doses of cycloheximide (CHX). Log phase cells were diluted and grown in YPD with or without CHX for 16-18 h, and then expression noise levels were determined. All measurements were conducted on early-log phase cells (0.3–0.5 OD_600_/ml). Both Tdh2-GFP and Adk1-mCherry exhibit increased expression noise upon drug treatment in a dosage-dependent manner (Kruskal–Wallis test, *P* = 4.464 × 10^−4^ for Tdh2-GFP and *P* = 4.356 × 10^−4^ for Adk1-mCherry, *n* = 6). Two-sided Mann–Whitney *U* test with Bonferroni correction was used for multiple comparisons and different letters above each column indicate statistically significant differences (adjusted *P*-value < 0.05). (**B**) Deleting *RPS9A*, *RPS28B* or *RPL42A*, but not *RPL22B*, in the WT background results in increased protein expression noise (Kruskal–Wallis test, *P* = 3.13 × 10^−5^ for Tdh2-GFP and *P* = 1.922 × 10^−5^ for Adk1-mCherry, *n* = 6). Every mutant was compared with WT (two-sided Mann–Whitney *U* test with Bonferroni correction). *, adjusted *P*-value < 0.05; ns, no significant difference. Horizontal solid lines represent median values of replicates. See Supplementary Table S6 for raw data and the details of statistical analysis.

Since our data reveals that Hos2 regulates both protein translation and protein expression noise, we tested if partially compromising the translation machinery could directly influence protein expression noise levels. To do so, we compared the noise levels of WT cells with or without translation inhibitor treatment. Upon treatment with a low dose of CHX that only mildly affects cell growth, we observed that the noise levels of the *TDH2-GFP* and *ADK1-mCherry* reporters significantly increased (Figure 4A). Moreover, protein expression noise became much more pronounced when the CHX dose was increased (Figure 4A).

Our transcriptomic analysis revealed that approximately one-third of ribosomal protein genes (40/137) were down-regulated in the *hos2*Δ mutant (Supplementary Table S4). After confirming the effect of translation inhibitors on protein expression noise, we investigated if mutating the Hos2 target genes would increase protein expression noise levels. We chose four non-essential ribosomal protein genes—*RPS9A*, *RPL22B*, *RPS28B* and *RPL42A*—the expression levels of which were strongly impacted by the *hos2* mutation (Supplementary Table S4). We observed that deleting *RPS9A*, *RPS28B* or *RPL42A* increased expression noise levels. Only the *rpl22b*Δ mutant did not exhibit a significantly noisy expression phenotype (Figure 4B). Several ribosomal subunits have redundant copies with overlapping functions, perhaps explaining why deleting some subunits does not affect noise regulation (64). Nonetheless, this finding suggests that the translation machinery is involved in regulating protein expression noise.

### *hos2*Δ cells exhibit strong fitness in a fluctuating environment

**Figure 5.**
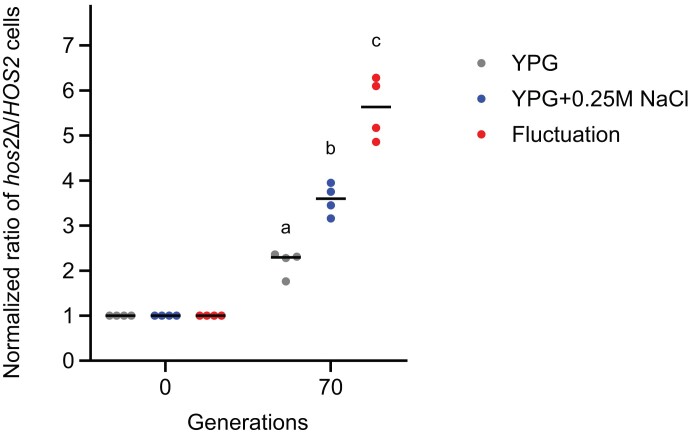
*hos2* deletion enhances cell competitiveness under a fluctuating environment. In the experimental set, WT and *hos2*Δ cells were mixed in a 1:1 ratio to start the competition assay under three growth conditions, i.e. constant YPG, constant YPG + 0.25 M NaCl, and periodic fluctuations between YPG and YPG + 0.25 M NaCl. A control set of cultures containing the same ratio of WT cells and ancestral cells with the selectable markers was grown in the same three conditions (see Materials and methods for details). Four independent cultures were set up for each condition. The *hos2*Δ-to-WT ratio was normalized to the control set ratio to control the marker gene effect. The normalized ratios of *hos2*Δ to WT cells are shown after 70 generations (One-way ANOVA, *P* = 1.106 × 10^−5^, *n* = 4). Tukey's HSD post-hoc test was used for multiple comparisons and different letters above each column indicate statistically significant differences (adjusted *P*-value < 0.05). Horizontal solid lines represent median values of replicates. See Supplementary Table S6 for raw data and the details of statistical analysis. See Supplementary Figure S5 for a time-course of altered fitness.

Expression noise could enable microorganisms to exert a ‘bet-hedging strategy’ to increase their competitiveness in fluctuating environments (15). We wondered if the *hos2* mutation, which increased protein expression noise, could endow advantages on a population growing in a non-optimal fluctuating environment. To explore that possibility, we performed a competition assay to measure how *hos2* cells competed with WT cells under growth conditions with or without osmotic stress and in a fluctuating environment. To create a fluctuating environment, we grew a mixed population of WT and *hos2*Δ cells (in a 1:1 ratio) in YPG with 0.25 M NaCl for 10 generations, and then propagated them in YPG alone for another 10 generations. We continuously switched the cells between these two conditions (i.e. YPG or YPG + 0.25 M NaCl) over the experimental course. In contrast, the other two cultures were grown constantly in either YPG or YPG + 0.25 M NaCl. Although *hos2*Δ cells displayed higher fitness than WT cells under all three conditions, they exhibited the highest *hos2*Δ-to-WT ratio in the fluctuating condition after 70 generations (Figure 5 and Supplementary Figure S4). This result suggests a connection between expression noise and advantageous impacts on a population during abrupt environmental shifts.

## Discussion

Protein expression noise can arise during many steps of gene expression, including at the transcriptional, post-transcriptional, translational and post-translational stages. Previous studies have revealed multiple molecular mechanisms by which transcriptional regulation modulates expression noise, but the role of translational regulation in noise control had remained elusive. We have uncovered that by regulating a specific group of ribosomal protein genes, the histone deacetylase Hos2 indirectly affects protein expression noise levels, revealing a close relationship between protein translation and noise control.

Hos2 is a member of the class I histone deacetylases (HDACs) that shares protein sequence similarity to Rpd3 (65). However, the cellular functions between Hos2 and Rpd3 are quite diverse. Rpd3 has the greatest effect on genome-wide deacetylation at gene promoters by acting as a general transcriptional repressor (61). In contrast, the targets of Hos2 are enriched for the open reading frames (ORFs) of ribosomal protein genes (61). Interestingly, Rpd3 was also found to be physically associated with ribosomal protein genes, but mainly in the promoter regions (66), implying that Rpd3 and Hos2 regulate gene expression in different ways. Deleting *RPD3* increases the frequency of transcription bursts, thereby reducing noise levels (25,67). In contrast, herein, we have identified Hos2 as a negative regulator of expression noise for proteins involved in distinct pathways (Figures 2B and 2D).

Our RNA-seq analysis shows that approximately one-third of ribosomal protein genes was down-regulated in the *hos2*Δ cells (Supplementary Table S4). Together with previous ChIP-on-chip data (61), this outcome indicates that Hos2 functions as a transcriptional activator of ribosomal protein genes. It has been suggested that once a gene is activated, the transcription machinery and histone acetyltransferases may disrupt the initial permissive state of chromatin, hindering further rounds of transcription. Deacetylation of the coding region by Hos2 can revert chromatin to the original permissive state, rendering it accessible for a new round of transcription (59).

Multiple lines of evidence in the current study support that increased expression noise in the *hos2*Δ mutant line is attributable to an indirect effect mediated through the Hos2-regulated translation machinery. First, our noise reporter genes, *TDH2* and *ADK1*, are not candidate Hos2-dependent DEGs (Supplementary Table S4) or Hos2 acetylation target genes (61). Second, both our polysome profiling and translation inhibitor sensitivity assays revealed that the translational machinery had changed in *hos2*Δ cells (Figures 3D-3F), and we have also shown that using translation inhibitors to compromise translation increased the expression noise of our reporter genes (Figure 4A). Third, when several Hos2-regulated ribosomal protein genes were deleted, the mutant cells exhibited high levels of expression noise (Figure 4B). Interestingly, a previous study showed that engineered changes in the abundance of a translation initiation factor, eIF4G, amplified protein expression noise in yeast (30), providing additional support for the role of protein translation in noise regulation.

How does the compromising translation machinery lead to increased protein expression noise? Recent quantitative analyses have revealed that translation is not a constitutive process in living cells. Similar to gene transcription, a single mRNA undergoes cycles between active and inactive states of protein translation, referred to as translation bursting (68–70). It has been suggested that 5’-cap proximal sequences and initiation factor dynamics contribute to the translation burst frequency, whereas secondary structures in the 5’-untranslated region (5’-UTR) of mRNAs affect burst amplitudes (70). Moreover, altering initiation factor abundance or 5’-UTR structures can influence noise levels (30,71). Since the 40S small ribosomal subunit forms part of the 43S pre-initiation complex, it is possible that reduced expression of small subunit genes in the *hos2*Δ mutant also impacts translation bursting and therefore translation noise.

Apart from the burst events during translation initiation, translational elongation may also contribute to protein expression noise. We observed increased protein expression noise levels in WT cells treated with mild doses of elongation inhibitors (Figure 4A). A low concentration of an elongation inhibitor only stalls some ribosomes, resulting in widespread collisions with uninhibited trailing ribosomes (72,73). Ribosome collisions can trigger the ribosome-associated quality control (RQC) pathway to rescue ribosomes and degrade any aberrant protein products (74,75). This process may contribute to fluctuations in protein translation and increase protein expression noise. Our *hos2*Δ cells also presented a high polysome-to-monosome ratio and an increased sensitivity to translation elongation inhibitors (Figure 3D–F), indicative of compromised translational elongation.

The effect of the Set3/Hos2 HDAC complex on noise regulation is much more obvious when cells are undergoing respiratory growth (Figure 2E), but that is not the case when cells are subjected to glucose-based growth conditions (25). This scenario is consistent with a previous observation that significant effects on expression noise levels were not detected for *set3* and *hos2* deletion mutants grown in a glucose medium (25). Given that the translational machinery is significantly down-regulated under respiratory growth (76), those conditions likely render cells more sensitive to reduced expression of their ribosomal genes. Interestingly, many slow growth conditions also reduce translation, which may sensitize the Hos2 noise regulation effect in a similar manner. Additional experiments are required to address this question of condition specificity.

In *Saccharomyces cerevisiae*, 59 of 78 ribosomal proteins are encoded by duplicate genes. Even though paralogous ribosomal proteins share high sequence similarity, individual deletions of these duplicates often elicit different fitness effects, some of which are condition-dependent (77,78). These observations indicate that there is a complex specialization of ribosomal proteins for specific cellular processes (79), which provides a possible explanation for why Hos2 only regulates a subset of ribosomal protein genes. It would be interesting to investigate if some ribosomal proteins contribute more to noise regulation than others.

Our experiments have shown that cell populations displaying high protein expression noise presented the best competitive performance in a fluctuating environment (Figure 5 and Supplementary Figure S4), further associating expression noise with beneficial effects in terms of short-term microbial competition. Notably, it has been suggested that noisy protein expression can enhance the phenotypic diversity of a population, leading to an increased survival rate under stress conditions (14). Many environmental stresses can reduce protein translation (80), which would probably amplify protein expression noise and influence long-term microbial evolution. However, increased expression noise can also impact cellular homeostasis and result in reduced fitness. Aged cells or organisms often exhibit high protein expression noise (81–84), indicating that their noise control system may have deteriorated. Our data indicate that Hos2 contributes to the expression noise control system. Moreover, Hos2 has been observed to form reversible cytosolic granules in quiescent yeast cells (85,86), adding another layer of regulation. To what extent is Hos2 regulation involved in the aging process and could modulating Hos2 affect lifespan? These are interesting questions that warrant investigation in the future.

## Supplementary Material

gkae432_Supplemental_Files

## Data Availability

The DNA-seq dataset is available at the NCBI BioProject database under accession PRJNA1023389. The RNA-seq data is available at the NCBI GEO database under accession GSE247734. All data are available in the main text or the supplementary materials.
