# Supplementary material for: Histone deacetylase Hos2 regulates protein expression noise by potentially modulating the protein translation machinery
Source: Nucleic Acids Res. 2024 May 23;52(13):7556–71. doi: 10.1093/nar/gkae432 (PMC11260488; doi:10.1093/nar/gkae432)
Supplement: gkae432_Supplemental_Files [file gkae432_supplemental_files.zip › Lin_et_al_supplementary_figure_R_0428.pdf]

A

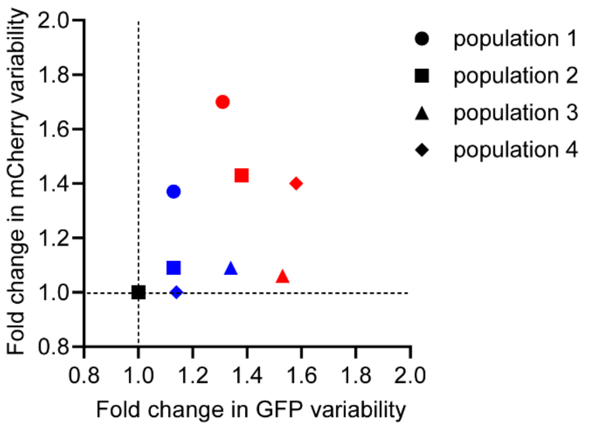

B

Evolved population	Reporters	Number of screened clones	Number of noisy clones	Name of evolved clones
Population 1	*ADK1-GFP*, *ADK1-mCherry*	34	0	-
Population 2	*ADK1-GFP*, *TDH2-mCherry*	32	1	Evo1
Population 3	*TDH2-GFP*, *ADK1-mCherry*	33	1	Evo2
Population 4	*TDH2-GFP*, *TDH2-mCherry*	32	3	Evo3, Evo4, Evo5

C

Evolved clone	Non-synonymous mutations	Synonymous mutations	Intergenic mutations	Total mutations
Evo1	21	5	12	38
Evo2	22	9	17	48
Evo3	21	5	12	38
Evo4	18	10	6	34
Evo5	30	9	15	54

Supplementary Figure S1. Experimental evolution to detect increased expression noise. (**A**) The protein abundance variability among cells of both reporters increase during experimental evolution. Four different ancestral strains were used to start the evolution experiment (Figures 1A and 1B). Blue and red data points represent the relative variability levels after the first and second rounds of selection, respectively. Data points with the same symbols denote populations derived from identical ancestral strains. (**B**) Details of the five evolved clones exhibiting increased expression noise after experimental evolution. For each evolved population, more than 30 individual clones were examined. Only clones that presented a consistently noisy phenotype were selected (see Materials and Methods). (**C**) Summary of the mutations in the five evolved clones. Non-synonymous mutations include missense, nonsense and frameshift mutations. Intergenic mutations indicate variants occurring upstream or

downstream of a coding region or in the intron. See also Supplementary Table S3 for details.

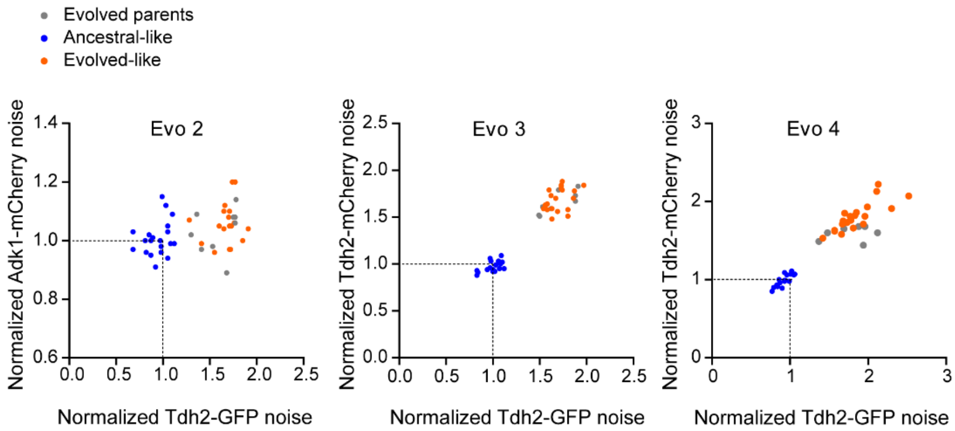

Supplementary Figure S2. Using bulk segregant analysis to identify causal variants in evolved clones. The relative noise levels in F1 progeny selected for whole-genome sequencing. To perform bulk segregant analysis, the evolved clones were backcrossed to their ancestral strains. For each cross, 64 individual F1 progeny derived from tetrad dissection were used to measure expression noise. A total of three replicates of protein expression noise measurement were conducted to classify progeny into ancestral-like (blue dots) or evolved-like (orange dots) bulks. For each bulk, 20 progenies with a constant phenotype were subjected to whole-genome sequencing. The relative fold-change in expression noise compared to the ancestral strain from the final run of analysis is shown. Gray dots represent the expression noise levels of the evolved parents. Since Evo2 mutations have less impact on Adk1-mCherry than on Tdh2-GFP (1.2-fold for Adk1-mCherry and 1.8-fold for Tdh2-GFP, Figure 1D), it makes the difference of Adk1-mCherry expression noise less detectable in the segregant pool. However, the *hos2-S215F* allele discovered in Evo2 does impact both Adk1-mCherry and Tdh2-GFP reporters (Figure 2B).

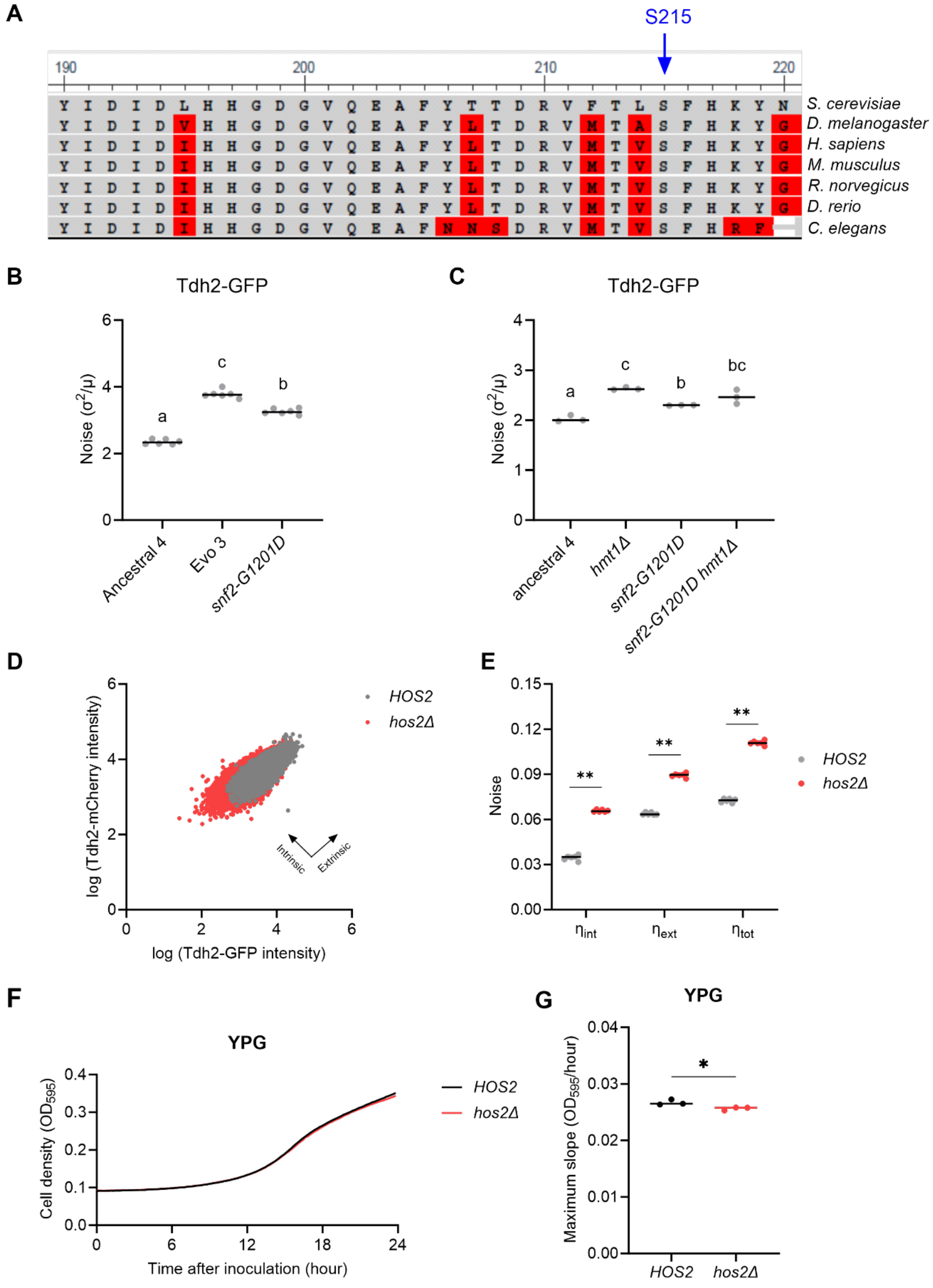

Supplementary Figure S3. Hos2 and Snf2 act as noise regulators. (**A**) The S215 residue in Hos2 is highly conserved among species. Amino acid sequence alignment of Hos2 (from residues 190 to 220 of *S. cerevisiae*) among different species. S215 is labeled with an arrow. (**B**) Reconstituting the *snf2-G1201D* mutation in the ancestral background increases Tdh2-GFP

expression noise levels. However, the expression noise level of *snf2-G1201D* is lower than Evo 3, indicating that other mutations are involved in the effect (Kruskal-Wallis test, $p = 4.922 \times 10^{-4}$, n = 6). Two-sided Mann-Whitney U test with Bonferroni correction was used for multiple comparisons and different letters above each column indicate statistically significant differences (adjusted *p*-value <0.05). (**C**) Genetic data indicate that Snf2 and Hmt1 likely act in the same pathway of protein expression noise regulation (One-way ANOVA, $p = 7.852 \times 10^{-5}$, n = 3). Tukey's HSD post-hoc test was used for multiple comparisons and different letters above each column indicate statistically significant differences (adjusted *p*-value <0.05). (**D**) The *hos2* mutant enhances both types of noise. Each data point represents the log-transformed mean fluorescence intensities of both Tdh2 reporters from a single cell. Spread of points perpendicular to the diagonal line on which GFP and mCherry intensities are equal corresponds to intrinsic noise, whereas spread parallel to this line is increased by extrinsic noise. (**E**) The intrinsic (η_{int}), extrinsic (η_{ext}) and total (η_{tot}) noise are calculated from Supplementary Figure S3D. **; p <0.01 (Two-sided Mann-Whitney U test, n = 6). (**F**) The growth kinetic curves of WT and *hos2* mutant cells during the early-log phase under the YPG condition were similar. (**G**) The *hos2* mutant causes a slight decrease in the maximum growth rate (~4%) under the YPG condition (Two-sided unpaired t-test with Welch's correction; $p = 0.0436$, n = 3). Horizontal solid lines represent median values of replicates. See Supplementary Table S6 for raw data and the details of statistical analysis.

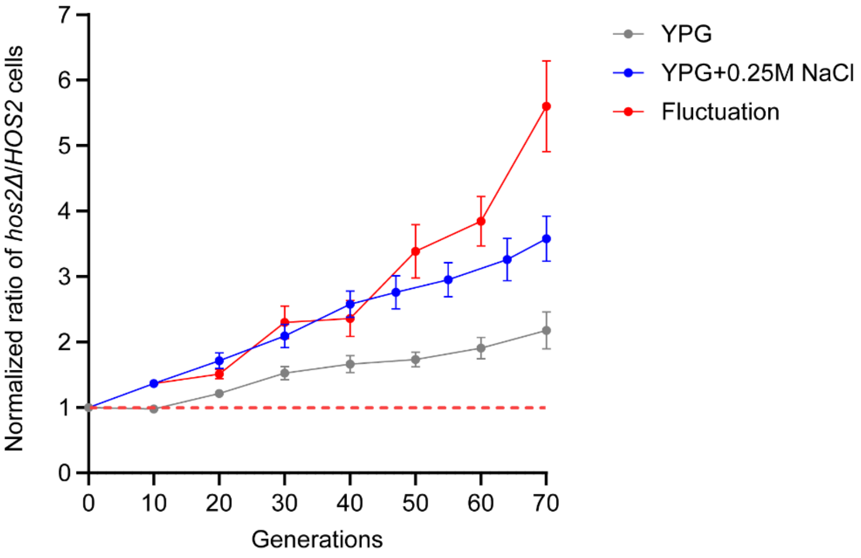

Supplementary Figure S4. *hos2*Δ exhibits strong competitiveness under a fluctuating environment. Relative fitness of the WT and *hos2*Δ strains was measured by competition assay under different growth conditions. Overnight cultures of competing strains were refreshed to early log phase and mixed in a 1:1 ratio (generation 0). The mixture was diluted and inoculated into distinct growth media. Cells were diluted into fresh media whenever the cell culture reached the early log phase. The ratio of *hos2*Δ to WT cells was calculated periodically and normalized to the ratio of ancestral to WT cells to control the marker gene effect.

Supplementary Table S1. Yeast strains used in this study.

Supplementary Table S2. Plasmids and primers used in this study.

Supplementary Table S3. List of mutations in the evolved clones.

Supplementary Table S4. List of the differentially expressed genes in the *hos2*Δ mutant.

Supplementary Table S5. Enriched GO terms in the *hos2*Δ mutant.

Supplementary Table S6. Raw data and statistical analyses.
